# The relationship of Fric test responses with an urticaria activity score, urticaria control test and quality of life scales in patients with symptomatic dermographism^؆^

**DOI:** 10.1016/j.abd.2025.501147

**Published:** 2025-07-07

**Authors:** Ömer Faruk Kıraç, Mustafa Tosun, Rukiye Yasak Güner, Melih Akyol

**Affiliations:** a Dermatology Department, Osmaniye Düziçi Hospital, Osmaniye, Turkey; b Dermatology Department, Sivas Cumhuriyet University Medicine Faculty, Sivas, Turkey

**Keywords:** Dermographism, Urticaria, Quality of Life

## Abstract

**Background:**

The FricTest is used as a valuable tool for diagnosing and conducting threshold testing for Symptomatic Dermographism (SD).

**Objective:**

In this study, the authors aimed to make a comparison between the Urticaria Activity Score (UAS), Urticaria Control Test (UCT), Visual Analog Scale (VAS), Dermatology Life Quality Index (DLQI), and Chronic Urticaria-Specific Quality of Life (CU-QoL) used to evaluate disease activity and control in the follow-up of urticaria patients and the Fric Test responses used in the diagnosis of dermographism.

**Methods:**

71 patients with SD were included in the study. Fric test 4.0 was performed in all patients at baseline and at month 1. The correlations of Fric test scores with UCT, UAS, VAS, DLQI, and CU-QoL at baseline as well as the changes in responses of treatment in the mean scores at month 1 were performed.

**Results:**

In the correlation analyses, positive correlations were observed between UAS, DLQI, and CU-QoL scores and changes in Fric test 4.5 mm and 4 mm responses from baseline to the first month of treatment (*p* < 0.05). No significant correlations were found between Fric test 3.5 mm and 3 mm responses and CU-QoL, UAS, DLQI, and VAS scores (*p* > 0.05).

**Study limitations:**

This study includes results from a small sample size, and larger-scale clinical trials are needed.

**Conclusion:**

Changes in the Fric test 4.5 mm and 4 mm responses of treatment were found to be more sensitive in detecting UCT, UAS, CU-QoL, and DLQI changes than the responses of the Fric test 3.5 mm and 3 mm.

## Introduction

Symptomatic dermographism (SD) is characterized by wheals that appear on the skin after pressure or trauma. The term's literal meaning is “writing on the skin”. Dermographism is observed in approximately 2–5% of the population. The most common form of chronic inducible urticaria (CIndU) is SD (dermographic urticaria, urticaria factitia), characterized by linear wheals with accompanying itching or sometimes burning and stinging sensations upon mild scratching or rubbing of the skin.^1^, ^2^, ^3^, ^4^ A provocation test using a dermographometer pen, the Fric test, or a blunt, smooth object like the tip of a ballpoint pen or wooden spatula confirms a diagnosis of SD based on the patient's history. The volar forearm or upper back skin is drawn with a blunt object for provocation, and the presence of wheals accompanied by itching, burning, and stinging sensations after ten minutes is positive.^5^

The urticaria activity score (UAS) is used to assess the severity of chronic urticaria based on the number of wheals and intensity of itching over the past week.^6^ The urticaria control test (UCT) assesses disease control in chronic urticaria patients over the last four weeks, focusing on symptoms, symptom control, impact on quality of life, and treatment effectiveness.^7^ The Chronic Urticaria-Specific Quality of Life (CU-QoL) assesses chronic urticaria's physical, daily, and psychosocial impacts over the past two weeks.^8^ The Dermatology Life Quality Index (DLQI) is a widely used, simple, and clear questionnaire that determines the impact of skin diseases and treatments on patients' symptoms, emotions, daily activities, leisure, work, school, and social interactions.^9^ Urticaria treatment comprises two main elements: the avoidance of triggering factors and medical treatment. Second-generation H1 antihistamines are the first-choice agents in pharmacological treatment, with omalizumab and cyclosporine forming the second and third stages of the disease control treatment algorithm, respectively.^1^, ^10^, ^11^, ^12^

This study aims to assess the relationship between Fric test responses, disease activity scores (UAS, UCT, and visual analog scale (VAS), and quality of life scales (CU-QoL and DLQI) in patients with SD. The authors investigated the potential of the Fric Test as a tool to assess disease severity and predict its impact on quality of life.

## Methods

The local Ethics Committee approved the present study (approval number: 2022-09/03, date: 20.09.2022). This study included 71 patients with SD who were followed up at the Hospital UCARE (Urticaria Centers of Reference and Excellence) between October 2022 and March 2023. The patients were using second-generation H1 antihistamines and omalizumab.

The authors obtained an informed consent form from all participants and informed them about the study. The authors excluded patients under the age of eighteen, those under immunosuppressive treatment, and those on systemic steroids. The authors recorded the demographic information of all participants.

The authors used UAS, UCT, and VAS to assess disease severity and control. In this study, the authors also used DLQI and CU-QoL to assess the impairment of quality of life (QoL).

### Design and evaluation of the study based on Fric test responses, urticaria activity score, urticaria control test, and quality of life tools

**Fig. 1:**
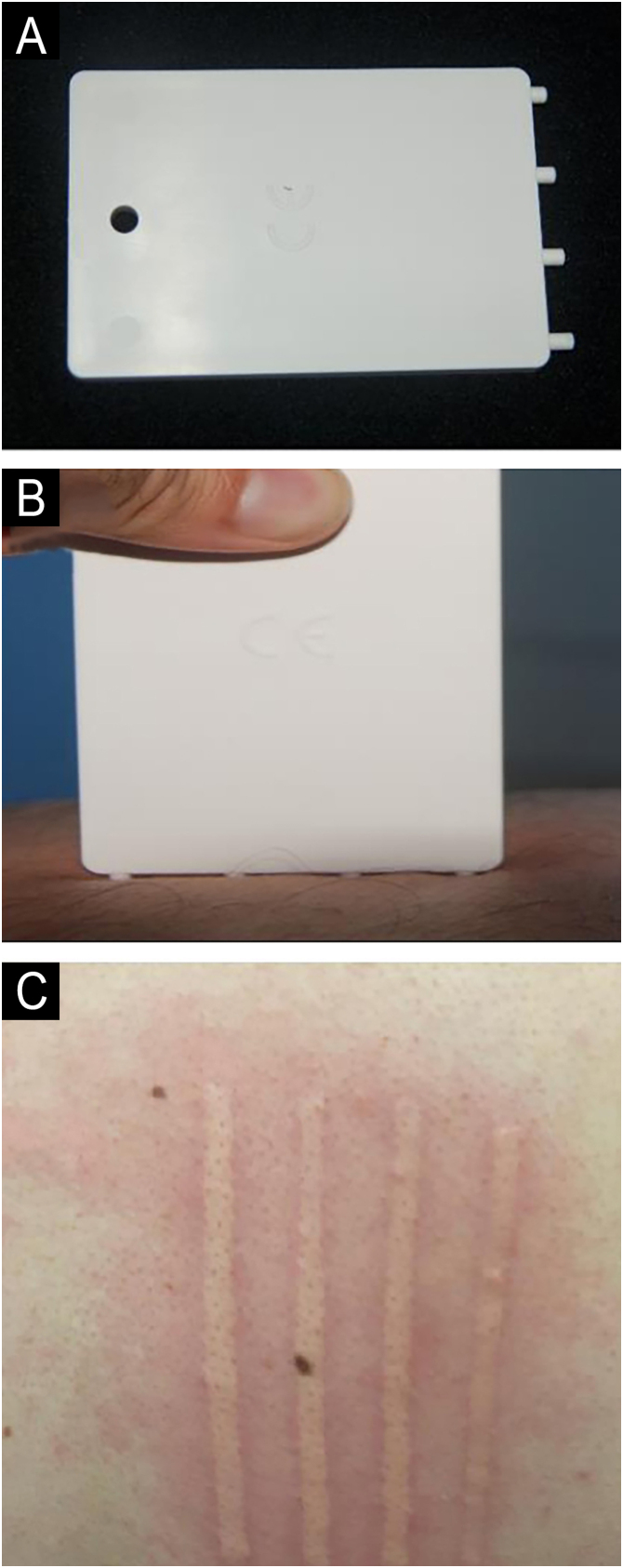
Fric test to determine dermographism. (A) Fric test 4.0. (B) Fric test applied perpendicular to the skin. (C) Wheals at the 10th minute as a result of the Fric test.

FricTest® 4.0 (Moxie, Germany) was applied perpendicular to the skin of the back with a pressure such that all pins (4.5 mm, 4 mm, 3.5 mm, and 3 mm) made complete contact (Fig. 1). The authors applied the Fric test to the back region of the patients in this study, both at baseline and during the first month of treatment. The authors measured the widths of the wheals formed at the 10th minute using a digital caliper within the four corners of the Fric test (4.5 mm, 4 mm, 3.5 mm, and 3 mm) and recorded them in centimeters. The authors calculated daily urticaria activity scores (UAS) at baseline and during the first month of treatment, summing the seven-day scores, to assess the severity of urticarial activity. The authors also applied the urticaria control test (UCT) at both baseline and the first month of treatment to assess the severity of the disease and the response to treatment.

Patients were asked to evaluate itching and burning complaints on a 10-point scale 10 minutes after the application of the Fric test in the first and 1-month examinations and recorded as VAS scores (10 points indicate the most severe itching and burning sensation, 0 points indicate no itching and burning sensation). The authors also administered the DLQI and the CU-QoL questionnaires at baseline and the first month of treatment to assess the impact of urticarial disease on daily activities, home, work, school life, social relationships, sleep, nutrition, personal care, and psychosocial status.

The authors analyzed correlations between the millimeter changes in Fric test responses from baseline to the first month and the changes in UAS, UCT, CU-QoL, DLQI, and VAS scores for the same period.

### Statistical analysis

The authors entered the collected data from this study into the SPSS software package (Version 22.0) for evaluation. The authors assessed the normality of the continuous data distribution using the Kolmogorov-Smirnov test. The authors provided mean, SD, median (min‒max), frequency, and percentages for quantitative variables as descriptive statistics. The authors used the Chi-Square test to compare categorical ratios. The authors used the Mann-Whitney *U* test to compare two independent groups and the Wilcoxon test to compare two dependent groups. The Spearman correlation test was applied. The authors set the level of significance (p-value) at 0.05.

## Results

The demographic data of patients with SD is in Table 1.

**Table 1:** Demographic data of patients with symptomatic dermographism (n = 71).

Age, years	
Min‒max (Median)	18‒75 (41)
Mean ± SD	41.5 ± 15.0
BMI, kg/m^2^	
Min‒max (Median)	14.2‒57.8 (26.4)
Mean ± SD	27.4 ± 6.6
Time of urticaria diagnosis (year)	
Min‒max (Median)	0.15‒21.0 (4)
Mean ± SD	5.8 ± 5.6
Gender	
Male	18 (25.4%)
Female	53 (74.6%)
Working status	
Not working	42 (59.16%)
Working	29 (40.84%)
Marital status	
Married	52 (73.24%)
Single	19 (26.76%)
Education level	
Primary Education	32 (45.07%)
Secondary Education	18 (25.35%)
Higher Education	21 (29.57%)
Smoking status	
No	54 (76.05%)
Yes	17 (23.9%)
Drinking status	
No	64 (90.14%)
Yes	7 (9.85%)

SD, Standard Deviation; BMI, Body Mass Index.

**Fig. 2:**
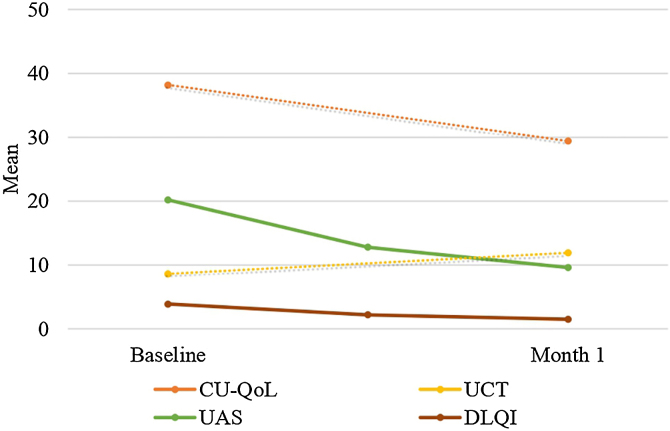
Changes in CU-QoL, UCT, UAS and DLQI during the treatment process.

**Fig. 3:**
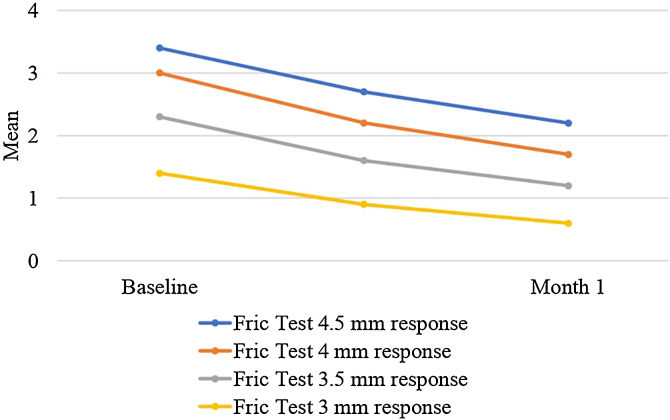
The change in responses of Fric test mm during the treatment process.

There was a statistically significant decrease in Fric test responses (4.5 mm, 4 mm, 3.5 mm, and 3 mm) from baseline to the first month of treatment (p < 0.05). Additionally, there was a statistically significant decrease in UAS, CU-QoL, DLQI, and VAS scores from baseline to the first month of treatment (p < 0.05). Furthermore, there was a statistically significant increase in UCT scores from baseline to the first month of treatment (p < 0.05) (Table 2, Fig. 2, Fig. 3).

**Table 2:** Comparisons of measurement parameters from baseline to the first month of treatment.

	Min‒max (Median)	*p*^a^
	Mean ± SD	
Baseline of CU-QoL	25‒74 (38)	<0.001
	38.2 ± 10.4	
Month 1 of CU-QoL	12‒42 (27)	
	29.4 ± 7.1	
Baseline of UCT	0‒16 (8)	<0.001
	8.6 ± 4.0	
Month 1 of UCT	4‒16 (12)	
	11.9 ± 2.7	
Baseline of UAS	3‒39 (21)	<0.001
	20.2 ± 8.8	
Month 1 of UAS	0‒28 (9)	
	9.6 ± 6.8	
Baseline of DLQI	0‒21 (3)	<0.001
	3.9 ± 3.4	
Month 1 of DLQI	0‒9 (1)	
	1.5 ± 1.2	
Baseline of Fric test 4.5 mm	1.6‒6.3 (3.2)	<0.001
	3.4 ± 1.0	
Month 1 of Fric test 4.5 mm	0‒4.6 (2.2)	
	2.2 ± 0.9	
Baseline of Fric test 4 mm	1.2‒5.6 (2.9)	<0.001
	3.0 ± 1.0	
Month 1 of Fric test 4 mm	0‒4.2 (2)	
	1.7 ± 1.1	
Baseline of Fric test 3.5 mm	0‒4.7 (2.4)	<0.001
	2.3 ± 1.3	
Month 1 of Fric test 3.5 mm	0‒4 (1.5)	
	1.2 ± 1.2	
Baseline of Fric test 3 mm	0‒4.2 (1.5)	<0.001
	1.4 ± 1.5	
Month 1 of Fric test 3 mm	0‒3.7 (0)	
	0.6 ± 1.1	
Baseline of VAS mm	0‒10 (4)	<0.001
	3.8 ± 2.8	
Month 1 of VAS	0‒10 (1)	
	2.1 ± 2.5	

CU-QoL, Chronic Urticaria Quality of Life Questionnaire; UAS, Urticaria Activity Score; UCT, Urticaria Control Test; DLQI, Dermatology Life Quality Index; VAS, Visual Analog Scale.

a The level of significance (p-value) was accepted as 0.05.

The Fric test 4.5 mm response was found to have a positive correlation with CU-QoL (*r* = 0.352, p = 0.003), UAS (*r* = 0.402, p = 0.001), and DLQI (*r* = 0.369, p = 0.002) from baseline to the first month of treatment. The Fric test 4.5 mm response was found to have a negative correlation with the UCT score (*r* = -0.398, p = 0.001). No statistically significant correlation was found between the Fric test 4.5 mm response and the VAS score (*r* = 0.180, p = 0.142). The Fric test 4 mm response was found to have a positive correlation with CU-QoL (*r* = 0.312, p = 0.009), UAS (*r* = 0.341, p = 0.004), and DLQI (*r* = 0.424, p < 0.001) from baseline to the first month of treatment. The Fric test 4 mm response was found to have a negative correlation with the UCT score (*r* = -0.397, p = 0.001). No statistically significant correlation was found between the Fric test 4 mm response and VAS score (*r* = 0.187, p = 0.130) (Table 3).

**Table 3:** Correlation analysis of changes in Fric test responses from baseline to the first month of treatment with different measurements.

Baseline to month 1 differences	R	p^a^
Fric test 4.5 mm response baseline to month 1		
CU-QoL	**0.352**	**0.003**
UCT	**-0.398**	**0.001**
UAS	**0.402**	**0.001**
DLQI	**0.369**	**0.002**
VAS	0.180	0.142
Fric test 4 mm response baseline to month 1		
CU-QoL	**0.312**	**0.009**
UCT	**.397**	**0.001**
UAS	**0.341**	**0.004**
DLQI	**0.424**	**<0.001**
VAS	0.187	0.130

CU-QoL, Chronic Urticaria Quality of Life Questionnaire; UCT, Urticaria Control Test; UAS, Urticaria Activity Score; DLQI, Dermatology Life Quality Index; VAS, Visual Analog Scale.

a The level of significance (p-value) was accepted as 0.05.

There was no statistically significant correlation between Fric test 3.5 mm response with CU-QoL (*r* = 0.097, p = 0.422), UAS (*r* = 0.196, p = 0.102), DLQI (*r* = 0.255, p = 0.052), UCT score (*r* = -0.194, p = 0.105), and VAS score (*r* = 0.010, p = 0.936) from baseline to the first month of treatment. Additionally, there was no statistically significant correlation between the Fric test 3 mm response and CU-QoL (*r* = 0.233, p = 0.052), UAS (*r* = 0.228, p = 0.058), DLQI (*r* = 0.152, p = 0.210), or VAS score (*r* = 0.083, p = 0.504) from baseline to the first month of treatment. There was only a statistically significant negative correlation between the Fric test 3 mm response and the UCT score (*r* = -0.344, p = 0.004) (Table 3).

## Discussion

SD is the most common subtype of chronic inducible urticaria, significantly limiting patients' daily functionality, work and school performance, and quality of life due to its psychosocial effects. For the diagnosis of SD, provocation tools such as a dermographometer pen, the Fric test, or a blunt-tipped, smooth object can be used. Schoepke et al. found the Fric test 4.0 to be 100% sensitive and 100% specific in diagnosing SD.^13^ The authors used the Fric test® 4.0 (Moxie, Germany) in the present study and recorded the corresponding values for 4 different tips (4.5 mm, 4 mm, 3.5 mm, and 3 mm) in millimeters. The authors investigated the correlation of these values with disease activity scores and quality of life scales.

In this study, the authors observed a significant positive correlation between the change in Fric test 4.5 mm response from baseline to the first month of treatment and changes in CU-QoL, UAS, and DLQI, and a significant negative correlation with the change in UCT. Similarly, the change in Fric test 4 mm response from baseline to the first month of treatment also showed a significant positive correlation with changes in CU-QoL, UAS, and DLQI, as well as a significant negative correlation with UCT change. However, the authors detected no significant relationship between the change in Fric test 4.5 mm and 4 mm responses from baseline to the first month of treatment and the VAS score.

No significant correlation was found between the 3.5 mm and 3 mm Fric test responses and changes in CU-QoL, UAS, VAS, and DLQI. Also, no significant relationship was identified between the change in the 3.5 mm Fric test response from baseline to the first month of treatment and the change in UCT score. However, a significant negative correlation was found between the change in the Fric test 3 mm response from baseline to the first month of treatment and the change in UCT score. Considering that an increase in UCT scores indicates improved urticaria control, the negative correlation between UCT score changes and Fric test responses is consistent with other findings in the present study.

Based on these results, it can be inferred that test results corresponding to 4.5 mm and 4 mm responses may provide more accurate information in predicting changes in CU-QoL, UCT, UAS, and DLQI, as well as in assessing disease activity and its impact on quality of life, compared to the responses for 3.5 mm and 3 mm ends.

Can et al. used the total Fric test score (TFS) in their study, which involved 58 patients and included initial visits and one-month follow-ups. In their study, changes in TFS showed a moderate positive correlation with changes in DLQI, and a significant negative correlation was found between changes in TFS and the physician's global assessment of disease control. However, in the same study, while the average changes in TFS showed a negative but non-significant correlation with changes in UCT, no correlation was found between changes in itching scores, the patient's global assessment of disease severity, and changes in TFS. The authors measured the reaction sizes for the four different ends of the Fric test in millimeters in this study, without using TFS; instead, the authors used the differences in millimeter measurements for correlation analysis. In the present study, the correlation of changes in the 4.5 mm and 4 mm ends of the Fric test from baseline to the first month of treatment with DLQI supports each other. Furthermore, the positive correlation of these changes with CU-QoL, UAS, and DLQI and the significant negative correlation with UCT indicate that the Total Fric Score's sensitivity to reflecting disease severity might be low. This suggests the need for recording measurements in millimeters or developing a new scoring method.^14^

In Ferreira et al.'s study comparing the quality of life of patients with chronic urticaria (CU) with its psychometric properties, CU-QoL was found to be highly correlated with DLQI, successfully differentiating between different severities of redness and itching.^15^ Although this study did not directly examine the relationship between these two measurement tools, it was observed that they correlate similarly with changes in Fric test responses. Particularly, the change in Fric test 4.5 mm and Fric test 4 mm responses from baseline to the first month of follow-up were found to be correlated with changes in DLQI and CU-QoL. This result supports the conclusion that the Fric 4.5 mm and 4 mm ends are more sensitive in patient follow-up.

Can and Kocatürk's study included patients with SD. The study validated treatment responses with increased UCT scores, physicians' global assessments of disease control, decreased DLQI scores, and patients's global assessments of disease severity. Omalizumab treatment was administered to 18 patients in the study, and by the 24^th^ week, the total response rates of patients had increased to 86%. Over time, UCT scores significantly increased, and VAS scores decreased.^16^

### Limitations

A limitation of this study is that it includes results from a small sample size, and larger-scale clinical trials are needed. Future studies assessing Fric test results more comprehensively could help us better understand patients' treatment responses and personalize treatment plans.

## Conclusion

In conclusion, the present study identified significant relationships between changes in Fric test results and changes in CU-QoL, UCT Score, UAS, and DLQI. In particular, test results corresponding to the 4.5 mm and 4 mm ends of the Fric test were found to be more sensitive in detecting changes in CU-QoL, UCT, UAS, and DLQI compared to responses from the 3.5 mm and 3 mm ends. There is a correlation between the Fric test results, particularly the 4.5 mm and 4 mm ends, and other clinical assessment measurements. This suggests that the Fric test could be a useful tool for assessing disease severity and predicting its impact on quality of life.

## Financial support

None declared.

## Authors' contributions

Ömer Faruk Kıraç: Writing, methodology, data curation, software, contributed to the clinical follow-up and management of patients.

Mustafa Tosun: Formal analysis, writing, approval of final manuscript, validation, visualization.

Rukiye Yasak Güner: Writing, methodology.

Melih Akyol: Formal analysis, and approval of the final manuscript.

## Conflicts of interest

None declared.
